# Hyaluronic acid production by *Klebsiella* *pneumoniae* strain H15 (OP354286) under different fermentation conditions

**DOI:** 10.1186/s12866-023-03035-0

**Published:** 2023-10-17

**Authors:** Rania M. Ahmed, Gamal Enan, Safaa Saed, Ahmed Askora

**Affiliations:** Department of Botany and Microbiology, Faculty of Science, Zagazig University, Zagazig, 44519 Egypt

**Keywords:** Cytotoxicity, Fermentation parameters, Hyaluronic acid (HA), *Klebsiella pneumonia*

## Abstract

**Background:**

Hyaluronic acid (HA) has gained significant attention due to its unique physical, chemical, and biological properties, making it widely used in various industries. This study aimed to screen bacterial isolates for HA production, characterize favorable fermentation conditions, and evaluate the inhibitory effect of bacterial HA on cancer cell lines.

**Results:**

A total of 108 bacterial isolates from diverse sources were screened for HA production using HPLC, turbidimetric, and carbazole determination methods. Among the HA-producing isolates, *Klebsiella pneumoniae* H15 isolated from an animal feces sample, was superior in HA production. The strain was characterized based on its morphological, cultural, and biochemical characteristics. Molecular identification using 16S rDNA sequencing and phylogenetic analysis confirmed its identity. Fermentation conditions, including pH, temperature, time, and agitation rate, were optimized to maximize HA production. The basal medium, comprising sucrose (7.0%) as carbon source and combined yeast extract with peptone (1.25% each) as nitrogen substrate, favored the highest HA production at pH 8.0, for 30 h, at 30 °C, under shaking at 180 rpm. The average maximized HA concentration reached 1.5 g L^−1^. Furthermore, bacterial HA exhibited a significant inhibitory effect on three cancer cell lines (MCF-7, HepG-2 and HCT), with the lowest concentration ranging from 0.98–3.91 µg mL^−1^.

**Conclusions:**

*K. pneumoniae* H15, isolated from animal feces demonstrated promising potential for HA production. The most favorable fermentation conditions led to a high HA production. The inhibitory effect of bacterial HA on cancer cell lines highlights its potential therapeutic applications. These findings contribute to a broader understanding and utilization of HA in various industries and therapeutic applications.

## Background

Hyaluronic acid, also called hyaluronan or hyaluronate (HA) is a linear, unbranched biopolymer composed of disaccharide repeats of *D*-glucuronic acid and *N*-acetylglucosamine which joined alternately by β-1,3 and β-1,4 glycosidic bonds. The molecular weights of HA are highly variable from different sources ranging from 10^4^ to 10^7^ Da comprising about 2,000–25,000 units [1]. Due to its unique physical, chemical, and biological properties such as viscoelasticity, distinctive moisturizing retention, high water-holding capacity, biocompatibility, wound healing, coupled with its lack of immunogenicity and toxicity, HA is widely used in several applications. These include drug manufacture, ophthalmology, rheumatology, healthcare, cosmetics, and the food industry [2–10]. HA was traditionally extracted from animal tissues. However, this method has limitations, such as the potential for cross-species viral infection and immunogenicity due to the use of hazardous solvents [11]. Additionally, animal-derived HA is complexed with proteoglycans, which is frequently associated with enzymes that degrade HA. Therefore, the use of microbial fermentation for HA production, offering lower production costs and reduced environmental pollution, is a pressing need [12, 13]. HA derived from polymers produced from bacteria was found identical with eukaryotic HA [14, 15]. The HA capsule is a natural virulence factor in immune system of some bacteria, such as *Streptococcus* sp. [16]. HA has been successfully produced in high productivity by *Streptococcus* spp., notably *S. equi*, *S. equisimilis*, *S. pyogenes* and *S. uberis* [13, 17]. However, the production of HA from *S. zooepidemicus* and *S. equisimilis* faces growing concern due to their pathogenicity [18]. In this regard, HA production with recombinant microorganisms such as *Bacillus subtilis*, *Lactococcus lactis*, *Escherichia coli*, *Agrobacterium* sp., *S. equi* subsp. *zooepidemicus* has attracted a considerable attention [1]. Culture conditions such as pH, temperature, agitation speed, aeration rate, shear stress, dissolved oxygen, and bioreactor type showed great effect on production of microbial metabolites, in general, and significantly influence the microbial HA production, in particular, [1, 19]. In literature, most studies have focused on production of HA by *Streptococcus* sp. Therefore, the search for new bacterial species capable of producing HA is an essential demand. Moreover, there is limited knowledge about the physiology of HA fermentation. From the technical, economic and environmental perspectives, it is desirable to develop high productivity of microbial fermentation of HA [20, 21]. Consequently, developing a cost-effective HA fermentation process using microorganisms is of paramount importance [22–26]. As a starting point in the current study, 108 bacterial isolates from various sources were screened for HA production. Nine isolates were positive for HA production and one promising strain of *Klebsiella pneumoniae* isolated from animal feces, was used as an attractive source for HA production in the current work. The most favorable fermentation conditions for enhanced HA production by this strain were explored. The potential use of HA as an antitumor agent was also investigated.

## Results

### Screening of bacterial strains for the production of HA

**Fig. 1:**
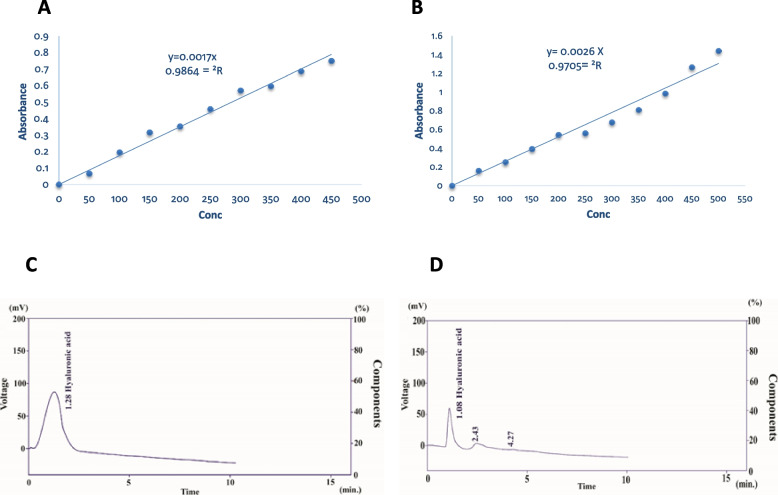
Qualitative and quantitative determination of HA produced by *K. pneumoniae* strain H15 using both carbazole and turbidimetric method. A standard curve was generated for carbazole (**A**) and turbidimetric (**B**) methods using a standard HA to quantify extracted HA in the bacterial supernatants. HPLC analysis confirmed the identity of HA separated from *K. pneumoniae* strain H15, exhibiting a peak with a retention time of 1.28 min that matched the authentic HA (**C**) under the same conditions, depicting the identical peak of HA separated from the crude extract of *K. pneumoniae* H15 (**D**)

Among 108 bacterial isolates recovered from different sources (soil, animal excretions, food and wound swab and blood samples from human subjects), nine isolates (8.33%) were found positive for HA production using a modified liquid medium. The isolates were screened for HA production using HPLC, turbidimetric and carbazole method. Using the standard HA, a standard curve of turbidity and carbazole method was plotted to quantify HA in the extracted bacterial supernatants (Fig. 1A and B). R^2^ indicating the functional linear relationship between HA concentration and the absorbance was above 0.986 for carbazole method and above 0.970 for turbidimetric method, as shown in the calibration graphs (Fig. 1A and B). The presence of HA in the crude extracts of the bacterial strains was confirmed by HPLC analysis of the HA-positive strains initially identified by VITEK MS, six strains of *K. pneumoniae* were dominant in HA production with varied potentiality (Table 1). *K. pneumoniae* H15 (isolated from animal feces sample) was the superior strain in HA production achieving 891, 932 and 940 mg HA L^−1^ using HPLC, turbidimetric and carbazole method, respectively; followed by *S. haemolyticus* HB9 (isolated from a patient blood sample) that produced 104, 176 and 232 mg HA L^−1^, respectively. *B. cereus* HW29 and *E. coli* AU5 showed a low HA-producing ability, compared with the two superior strains. Using HPLC determination, the first strain (HW29 isolated from a patient wound sample) showed a sharp less HA potentiality by 15.63 folds than that of H15 strain. The second strain (AU5 isolated from animal urine) produced HA in an amount less than that of H15 strain by 19.8 folds. The lowest HA-producing ability was obtained by *K. pneumoniae* CS22 (isolated from a cultivated soil) giving 8.00 mg HA L^−1^(Table 1).The HPLC analysis confirms the identity of HA separated from the bacterial cultures showing a peak with a retention time of 1.28 min that matched the authentic HA under the same conditions (Fig. 1C and D shows identical peak of HA separated from the crude extract of *K. pneumoniae* H15, the most active isolate with the authentic HA). Therefore, *K. pneumoniae* H15 was used as experimental organism throughout this study in order to determine some favorable fermentation conditions that may contribute to improving HA production.

**Table 1:** HA-producing bacterial strains identified by VITEK MS

HA-producing bacterial strains	Code no	Source of isolation	Concentrations of HA (mg L^−1^)		
			HPLC method	Turbidity method	Carbazole method
*K. pneumoniae*	CS3	Cultivated soil	90.0 ± 20.0^**cd**^	127 ± 24.0^**bc**^	125 ± 20^**bc**^
*K. pneumoniae*	CS22	Cultivated soil	8.00 ± 1.00^**e**^	15.0 ± 3.00^**e**^	15.0 ± 1.00^**e**^
*K. pneumoniae*	AU3	Animal urine	67.0 ± 14.0^**cd**^	92.0 ± 1.00^**bc**^	128 ± 41.0^**bc**^
*E. coli*	AU5	Animal urine	45.0 ± 15.0^**d**^	85.0 ± 14.0^**cd**^	88.0 ± 11.0^**cd**^
*K. pneumoniae*	H15	Animal faces	891 ± 28.0^**a**^	932 ± 20.0^**a**^	940 ± 32.0^**a**^
*S. haemolyticus*	HB9	Patient blood	104 ± 27.0^**cd**^	176 ± 33.0^**cd**^	232 ± 53.0^**bc**^
*K. pneumoniae*	HW16	Patient wound	6.00 ± 1.00^**e**^	14.0 ± 2.00^**e**^	13.0 ± 1.00^**e**^
*K. pneumoniae*	HW24	Patient wound	14.0 ± 1.00^**e**^	45.0 ± 3.00^**d**^	44.0 ± 3.00^**d**^
*B. cereus*	HW29	Patient wound	57.0 ± 1.00^**d**^	98.0 ± 3.00^**cd**^	102 ± 3.00^**cd**^

Calculated mean is for triplicate measurements from two independent experiments ± SD; means with different superscripts in the same column are considered statistically different (LSD test, *P* ≤ 0.05)

Different letters on the bars (a, b, c, d, e) indicate significant differences between the data points

### Morphological and molecular identification of H15 isolate

**Fig. 2:**
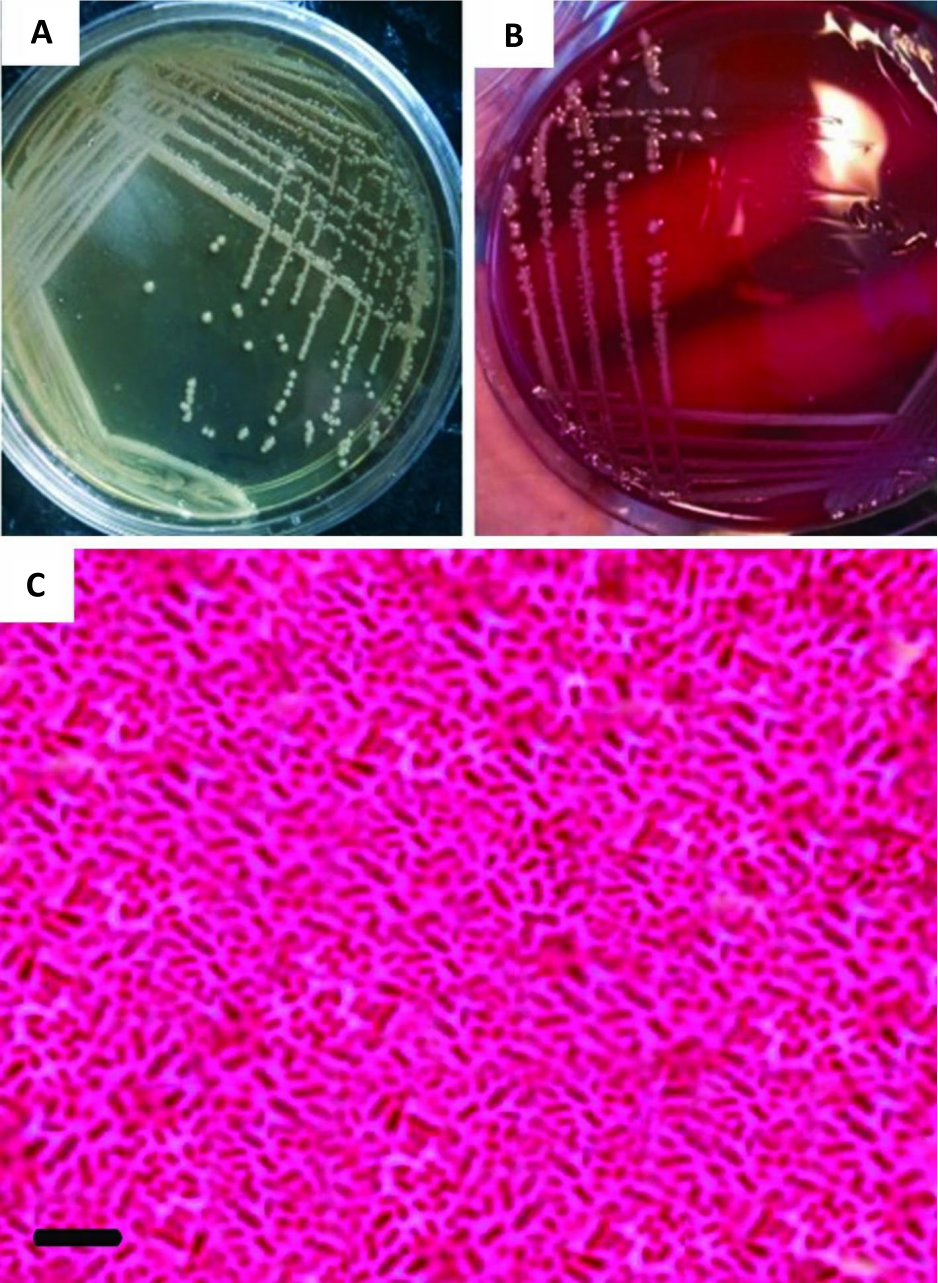
Morphology of *K. pneumoniae* strain H15 colonies on selective differential media. **A** Brain Heart Infusion (BHI) agar medium with mucoid, and greyish white colonies appearance, and **B** Blood agar with non-hemolytic colonies with a diameter about3-4 mm. **C** Microscopic examination of the isolate showing Gram-negative stain with short rod morphology and non-spore forming characteristics (scale bar = 0.95 µm)

**Fig. 3:**
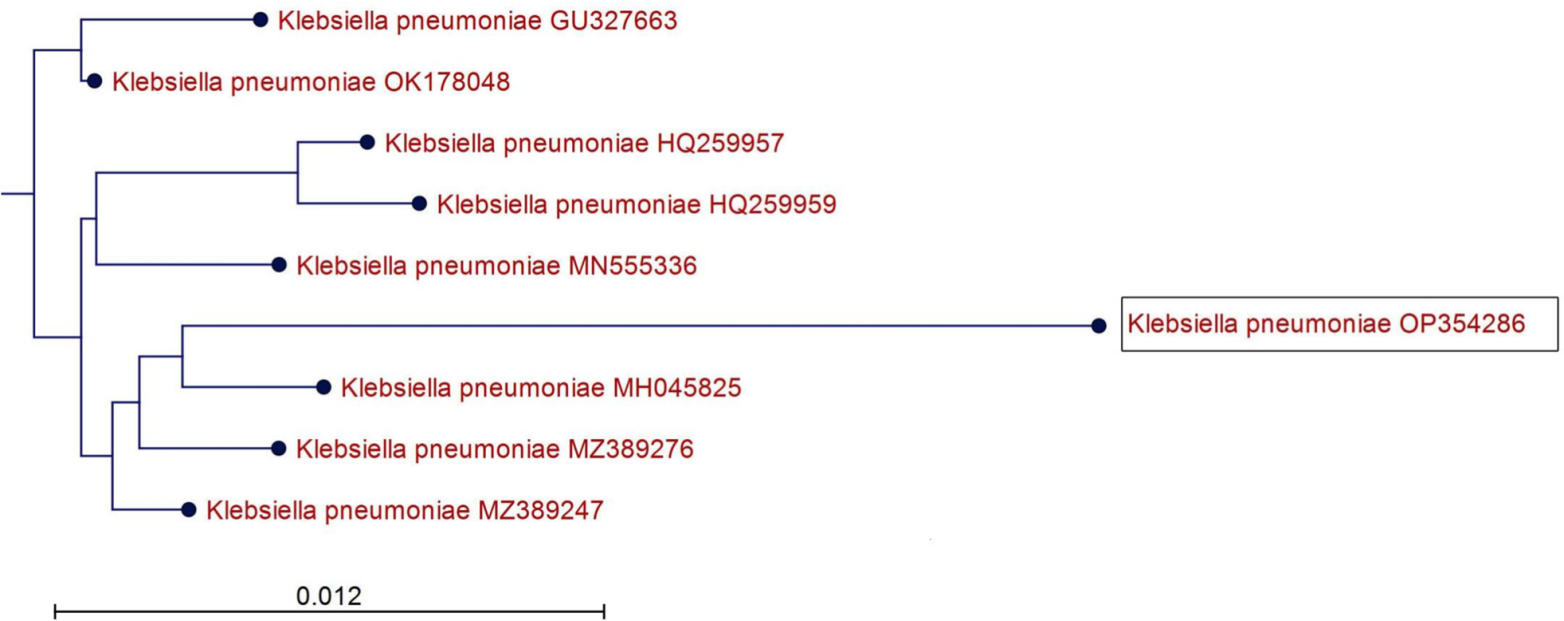
Phylogenetic tree analysis based on 16S rRNA nucleotide sequence alignment for *K. pneumoniae* strain H15 with other related members that possess the best similarity. The scale bar at the bottom (left) indicates similarity coefficient (%)

This isolate H15 was subjected to cultural, morphological and molecular characterization. The cultural characteristics were observed when streaked on BHI gar and blood agar. After incubation at 37ºC for 24 h, it appeared as large, opaque, mucoid, and greyish white colonies on BHI agar (Fig. 2A). On blood agar surface, colonies are non-hemolytic with a diameter about 3–4 mm (Fig. 2B). Microscopic examination showed that the isolate is Gram-negative short rod and non-spore forming (Fig. 2C). The H15 isolate was molecularly identified via extraction of the genomic DNA and amplification of rRNA by PCR. Using 16S rRNA sequence data, the phylogenetic analysis of the bacterial strain H15 suggested that this strain is closely related to *K. pneumoniae* and it was deposited in the GenBank under accession number OP354286. The 1104 bp partial sequence of 16S rRNA was aligned to construct a phylogenetic tree (Fig. 3), and alignment data showed a 98% identity with *K. pneumoniae* accession numbers MZ389276, and MZ389247, OK178048.

### Effect of cultural conditions on the production of HA by *K. pneumoniae* H15

#### Effect of initial pH

**Fig. 4:**
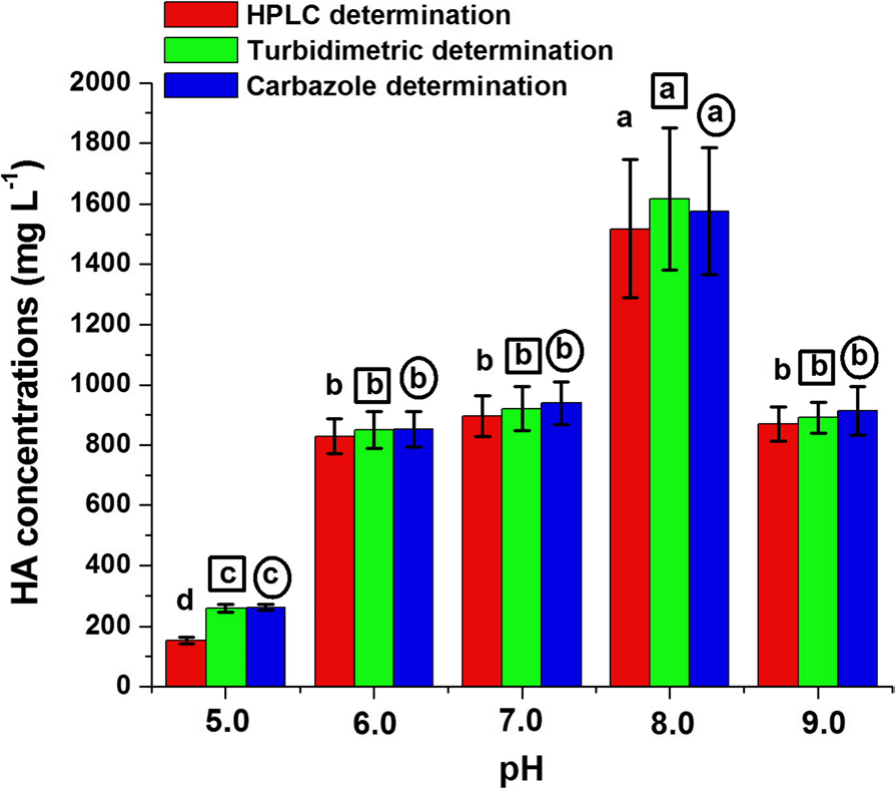
Effect of pH on HA production by strain H15 in the modified medium. The production of HA was assessed at various pH values by adjusting the medium with 1 N HCl and 1 N NaOH. Values are shown ± SD of triplicate measurements from two independent experiments. Different letters on the bars of HA concentrations determined by HPLC method (free letters), turbidimetric method (in small squares) and carbazole method (in small circles) indicate significant differences (LSD, *P* ≤ 0.05) at different pH values

The production of HA by the strain H15 was analyzed at different initial pH values of the modified medium using varying amounts of 1 N HCl and 1 N NaOH. Results showed that the final pH values of the culture medium were in between 3.5 and 4.3. The HA production was promoted at the alkaline initial pH values, and the maximum HA concentrations were significantly obtained at pH 8.0 recording 1517, 1616 and 1575 mg L^−1^ by HPLC, turbidimetric and carbazole method, respectively (Fig. 4). Considerable HA concentrations of 869, 891 and 913 mg L^−1^ were also obtained by the three respective determinations at the highly alkaline initial pH 9.0. Comparable HA concentrations were produced by the experimental strain at initial pH 6.0 and 7.0. Decreasing the initial pH value to the acidic side (pH 5.0) attained sharp decreases in HA production.

#### Effect of incubation temperature

**Fig. 5:**
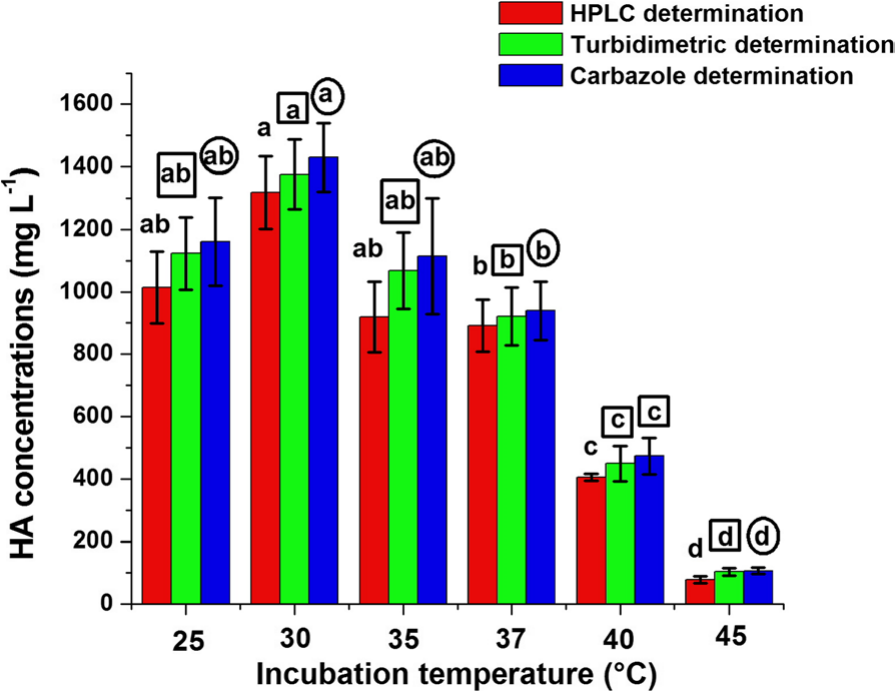
Effect of incubation temperature on HA production by strain H15. Values are shown ± SD of triplicate measurements from two independent experiments. Different letters on the bars of HA concentrations determined by HPLC method (free letters), turbidimetric method (in small squares) and carbazole method (in small circles) indicate significant differences (LSD, *P* ≤ 0.05) at different temperatures

Data in Fig. 5 indicated that the HA biosynthesis by the experimental organism was greatly influenced by incubation temperature. Maximum HA production was obtained at 30 °C recording 1318, 1376 and 1430 mg L^−1^using HPLC, turbidimetric and carbazole determination, respectively. At this temperature, significant difference in HA concentrations (*P* ≤ 0.05) was obtained when HA productions by the determination methods were compared separately at temperatures tried. Satisfactory concentrations of HA were obtained at 25 °C achieving 76.93, 81.61 and 81.12% of HA concentrations produced by the respective three determinations at 30 °C. Fermentation conducted at higher temperatures (35 to 45 °C) promoted gradual decrease in HA concentrations.

#### Effect of fermentation time

The effect of time course profile on HA production by the experimental strain was also studied (Table 2). Data obtained showed that HA biosynthesis was increased with increase in the fermentation period till achieving the highest concentration of 1352, 1474 and 1448 mg HA L^−1^ at 30 h of fermentation by HPLC, turbidimetric and carbazole determination, respectively. After which, the HA concentrations were decreased upon increasing the incubation time at 36 h recording1222, 1266 and 1267 mg HA L^−1^.

**Table 2:** Effect of fermentation times on HA production by AF17 strain

Fermentation time (h)	Concentrations of HA (mg L^−1^)		
	HPLC method	Turbidity method	Carbazole method
12	748 ± 58.0^c^	791 ± 64.0^c^	808 ± 51.0^c^
18	860 ± 62.0^c^	882 ± 113^bc^	905 ± 104^bc^
24	903 ± 116^bc^	918 ± 171^bc^	948 ± 88.0^bc^
30	1352 ± 116^a^	1474 ± 156^a^	1448 ± 115^a^
36	1222 ± 68.0^ab^	1266 ± 164^ab^	1267 ± 91.0^ab^

Calculated mean is for triplicate measurements from two independent experiments ± SD; means with different superscripts in the same column are considered statistically different (LSD test, *P* ≤ 0.05)

Different letters on the bars (a, b, c) indicate significant differences between the data points. Bars with the same letter are not significantly different, while bars with different letters are

#### Effect of agitation rate

**Fig. 6:**
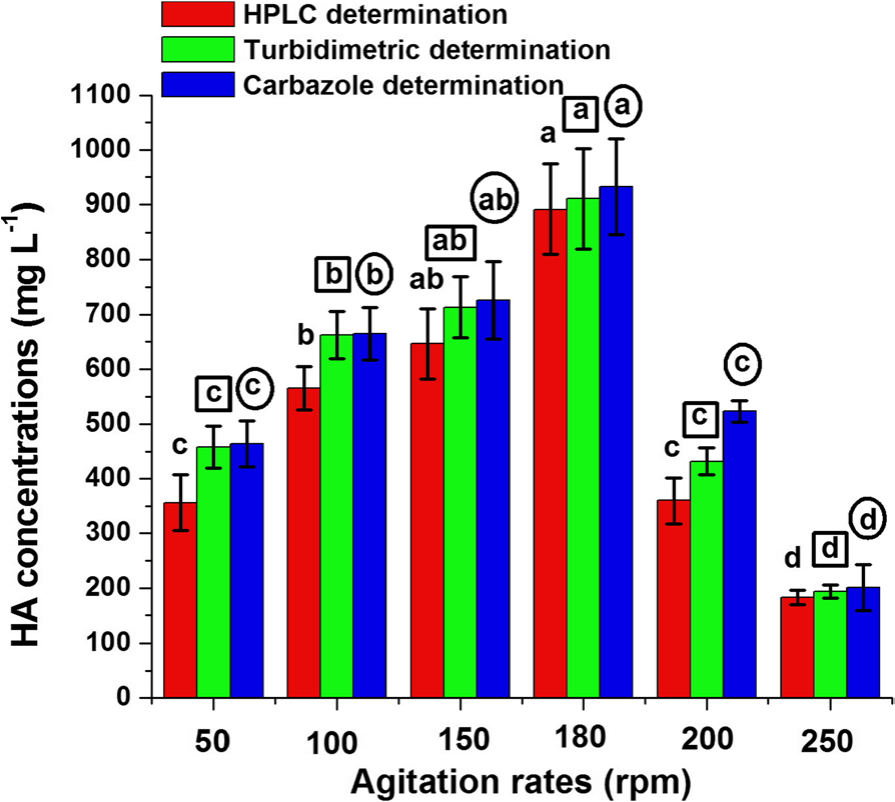
Effect of agitation rate on HA production by strain H15. Values are shown ± SD of triplicate measurements from two independent experiments. Different letters on the bars of HA concentrations determined by HPLC method (free letters), turbidimetric method (in small squares) and carbazole method (in small circles) indicate significant differences (LSD, *P* ≤ 0.05) at different agitation rates

The production of HA by *K. pneumoniae* H15 was further analyzed at different agitation rates (Fig. 6). The highest HA concentration was achieved at 180 rpm (the control agitation rate) recording 892, 911 and 933 mg L^−1^ using HPLC, turbidimetric and carbazole determination, respectively. At this agitation rate, significant difference (*P* ≤ 0.05) in HA concentration was obtained by the three methods of determination, when compared separately. After which, the HA concentration was gradually decreased by increasing the agitation rate. Noteworthy, the agitation at the lowest rate (50 rpm) promoted higher HA concentration than that obtained by the highest agitation rate (250 rpm) using the three HA determinations. At 50 rpm, the HA production was 1.94, 2.36 and 2.31 folds of that obtained at 250 rpm by the three respective determinations.

#### Effect of C and N sources

To elucidate the impact of nutritional sources on HA production by the experimental strain, we tested various carbon (C) and nitrogen (N) sources in the basal medium. Sucrose, used as the control carbon source, yielded the highest HA concentrations: 889, 919, and 941 mg HA L^−1^ as determined by HPLC, turbidimetric, and carbazole methods respectively (Table 3). Fructose and glucose produced comparable HA levels with differences from sucrose being statistically insignificant (*P* ≥ 0.05). Their yields ranged from 751 to 854 mg HA L^−1^ across the three determination methods. In contrast, lactose and maltose proved to be less effective. Lactose-derived HA was 30.5 to 38.8% of sucrose-derived HA, depending on the determination method. For maltose, this decrease ranged between 41.1% and 47.8%. The combination of yeast extract with peptone (used as the control N source) yielded the highest HA levels: 892, 924, and 956 mg HA L^−1^ across the three determination methods (Table 3). Using only yeast extract as the nitrogen source, HA concentrations were comparable to the combination, with levels ranging from 751 to 878 mg HA L-1 and no significant difference (*P* ≥ 0.05). Solely using peptone resulted in HA concentrations representing 62.2% to 71.5% of those achieved with the yeast extract with peptone combination.

**Table 3:** HA production by H15 strain under different C and N sources

C and N sources	Concentrations of HA (mg L^−1^)		
	HPLC method	Turbidity method	Carbazole method
Glucose	751 ± 29.0^a^	785 ± 27.0^a^	796 ± 64.0^a^
Sucrose	889 ± 49.0^a^	919 ± 63.0^a^	941 ± 93.0^a^
Lactose	544 ± 21.0^c^	627 ± 30.0^c^	654 ± 27.0^c^
Maltose	464 ± 41.0^c^	531 ± 52.0^c^	554 ± 44.0^c^
Fructose	772 ± 23.0^a^	843 ± 44.0^a^	854 ± 52.0^a^
Peptone	555 ± 40.0^b^	661 ± 29.0^b^	683 ± 37.0^b^
Yeast extract	751 ± 34.0^a^	861 ± 37.0^a^	878 ± 41.0^a^
Yeast extract + peptone	892 ± 36.0^a^	924 ± 44.0^a^	956 ± 45.0^a^
Ammonium sulfate	000 ± 000	000 ± 000	000 ± 000
Sodium nitrate	000 ± 000	000 ± 000	000 ± 000

Calculated mean is for triplicate measurements from two independent experiments ± SD; means with different superscripts in the same column either for C or N sources are considered statistically different (LSD test, *P* ≤ 0.05). In the case of testing C sources, the cultivation medium contained combination of yeast extract and peptone as N source. In the case of testing N sources, the cultivation medium contained sucrose as C source

Different letters on the bars (a, b, c) indicate significant differences between the data points. Bars with the same letter are not significantly different, while bars with different letters are

Interestingly, using inorganic nitrogen substrates led to undetectable HA concentrations, emphasizing the importance of organic complex N substrates for HA biosynthesis.

#### Cytotoxicity of HA

**Fig. 7:**
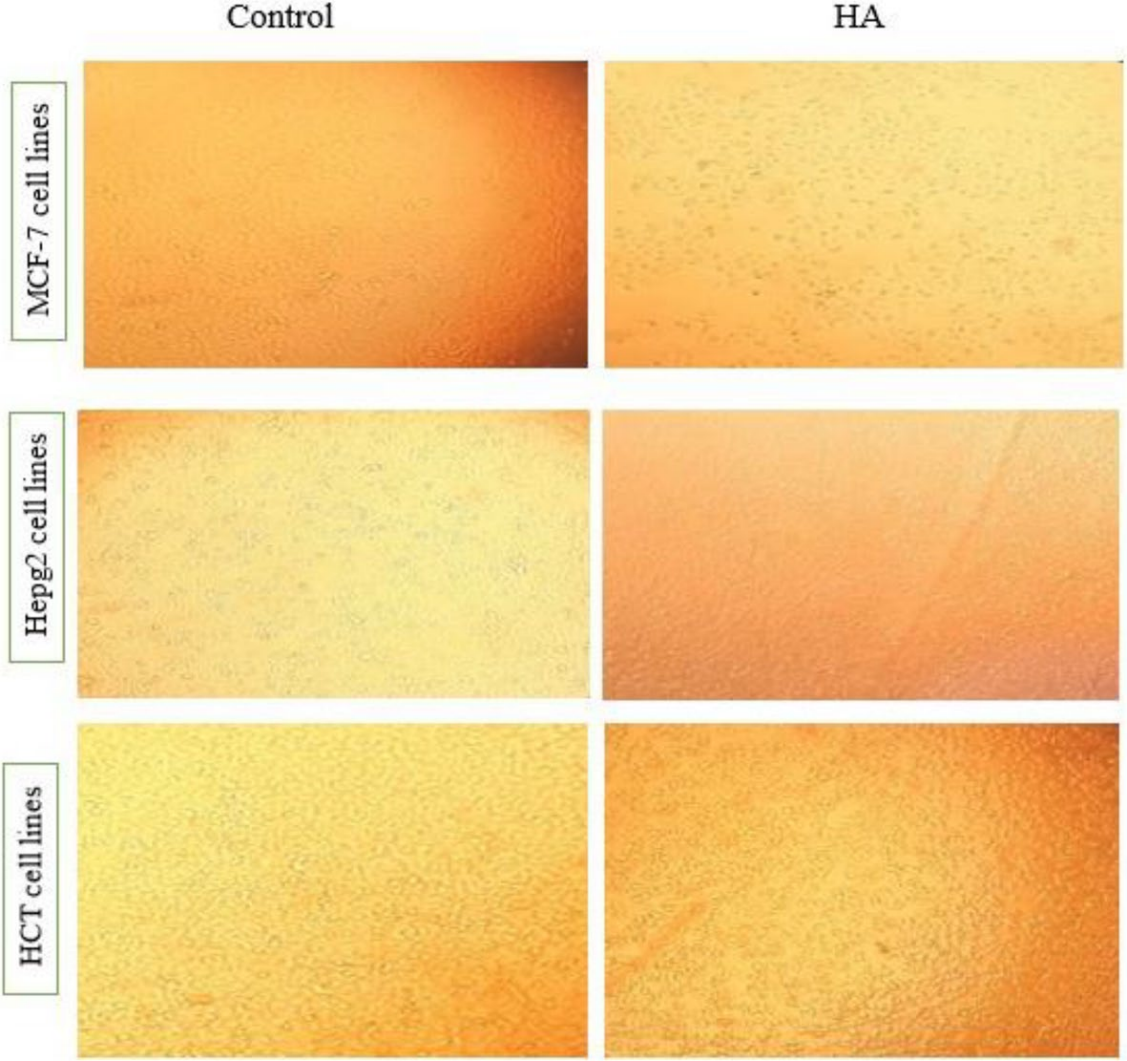
Cytotoxicity of HA against MCF-7, HepG-2, and HCT cell lines. The cytotoxic effects of HA were evaluated against three different cell lines; MCF-7 (breast), HepG-2 (liver), and HCT (colon). Various concentrations of HA were tested. The data revealed that HA exhibited the lowest inhibitory concentration of 0.98 µg mL^−1^ against both MCF-7 and HCT cells, while it was 3.91 µg mL^−1^ against HepG-2 cells

The cytotoxicity of HA was demonstrated against three cancer cell lines: viz. MCF-7 (breast), HepG-2 (liver), and HCT (colon) at various concentrations (Table 4, Fig. 7). Fig. 7 presents the cytotoxic effects of HA on the three cancer cell lines: MCF-7, HepG-2, and HCT. The vulnerability of each cell line varies with HA concentrations. MCF-7 and HCT cells show significant inhibitory effects at a concentration of 0.98 µg mL^−1^, while HepG-2 cells require 3.91 µg mL^−1^. As the concentration of HA increased, there was a consistent decline in the proliferation rates of all cancer cell lines. Diverse IC50 values and viability percentages highlight the distinct sensitivities of these cell lines to HA. Notably, HCT cells appear to be more susceptible than the others. In Table 4, the IC50 values, which indicate the concentration at which 50% of cells are inhibited, for MCF-7 and HepG-2 are 1538 µg mL^−1^ and 1209 µg mL^−1^, respectively. At 500 µg HA mL^−1^ (the higher concentration tested), the cell viability recorded was 57.4% against MCF-7, 63.3% against HepG-2, and 27.8% against HCT.

**Table 4:** Anticancer activity of HA against three cancer cell lines

HA conc. (µg mL^-1^)	Cell viability (%)		
	MCF-7 (breast)	HepG-2 (liver)	HCT (colon)
500	57.40± 1.96^e^	63.33±2.41^d^	27.83±6.93^d^
250	64.69± 1.21^d^	64.59±1.66^d^	58.00±2.63^c^
125	74.58± 5.21^cd^	72.52±1.85^c^	75.33±3.74^b^
62.5	74.69± 7.25^c^	73.19±2.11^c^	77.83±4.11^b^
31.25	76.88± 3.96^c^	76.67±3.33^bc^	77.83±3.62^b^
15.63	80.52± 5.24^bc^	81.85±1.17^b^	78.33±2.22^b^
7.81	89.69± 1.62^b^	93.78±2.86^a^	79.00±4.62^b^
3.91	95.10 ±2.82^ab^	99.63±3.10^a^	79.33±3.85^b^
1.95	98.33 ±1.66^a^	100±5.23^a^	84.17±2.71^b^
0.98	99.48 ±2.17^a^	100±3.62^a^	98.33±2.46^a^
0.49	100±1.29^a^	100±4.22^a^	100±3.82^a^
0.24	100±2.33^a^	100±2.63^a^	100±6.23^a^
IC_50_ (µg mL^-1^)	1538.06	1209.21	273.40

Means± SD in the same column with different superscripts are significantly different (LSD test, *P* ≤ 0.05)

Different letters on the bars (a, b, c, d, e) indicate significant differences between the data points

## Discussion

Microbial fermentative production of biomolecules, particularly biodegradable polymers like HA, has garnered global interest due to their eco-friendly nature and wide-ranging applications in pharmaceuticals, medical fields, biomaterials, and cosmetics [27, 28]. These biopolymers are favored for their viscoelasticity, biocompatibility, water-retention, and non-immunogenic properties. Furthermore, they do not generate toxic degradation products [29]. Commercially, HA production occurs by two approaches. The first process involves extraction from the tissues of rooster combs, which faces challenges in extraction, possible degradation, high purification costs, and potential contamination risks like viruses [30]. The second process is employed through fermentation by Streptococcus strains, which yields HA that is both non-immunogenic and biocompatible [31]. However, the use of Streptococcus poses a challenge due to its potential to produce exotoxins. [1]. This has led to innovations such as producing HA from cell-free systems to avoid endotoxin contamination and reduce purification costs [18, 32]. This study aimed to identify new bacterial isolates for enhanced HA production. Out of 108 bacterial isolates screened, nine were found capable of HA production, as identified by VITEK MS. These were predominantly *K. pneumoniae*, followed by single isolates of *E. coli*, *S. haemolyticus*, and *B. cereus*. HA concentration assessments utilized HPLC, turbidimetric, and carbazole methods. The HPLC results highlighted clear HA peaks without interference. Carbazole quantification, while detailed, is deemed tedious due to its sulfuric acid reliance, making turbidimetric, which involves HA-CTAB complex formations, a faster alternative [33, 34]. The HPLC technique by Çağlar differentiated HA at 7.54 min, proving its heightened accuracy over other methods [35, 36]. In this context, *K. pneumoniae* H15 stood out for it's HA production capacity of 891 mg L^−1^, echoing findings from another study [37]. As addressed previously, we applied three different methods for HA determination to ensure a comprehensive understanding and validation of our results, as well as to present a comparative study on the efficiency of these methods. Certainly, addressing the choice to use the carbazole method for HA determination despite its known labor-intensive and time-consuming nature requires a comprehensive justification. The carbazole method, while being laborious, is a well-established and traditional method for HA determination. By employing this method alongside newer or alternative techniques, it provides a reference or benchmark. This can help validate the results from other methods, ensuring accuracy and reliability. While the carbazole method has its drawbacks in terms of the time and effort required, its benefits in terms of accuracy, specificity, and wide acceptance often make it a method of choice, especially when used in combination with other methods for a holistic understanding of HA concentrations. Historically, main HA producers are Streptococci, notably *S. equi* and *S. zooepidemicus* [1, 28, 38–41], but modified bacterial species and an engineered *E. coli* strain have also been documented [42]. Morphologically, *K. pneumoniae* H15 conforms to prior research when cultured on BHI agar and blood agar [43–45]. Its identification, executed through the VITEK 2 system, was further solidified by 16S rRNA sequencing and a 98% identity match with specific *K. pneumonia* strains in GenBank. The resultant phylogenetic tree aligned coherently with universally acknowledged species definitions based on culture and morphology [43, 46]. This study sought to determine the influence of various cultivation conditions on HA production by *K. pneumoniae* H15. Our findings show that the best HA production levels were achieved at a pH of 8.0, highlighting a preference for alkaline conditions. Similarly, Pan [47] found optimal HA production by *Streptococcus zooepidemicus* at this pH. Ucm [28] and Chen [41] reported similar trends, emphasizing that a pH closer to 8.0 was ideal. Conversely, other research found pH levels ranging from 6.7 to 7.8 to be more favorable [17, 48, 49]**.** The temperature had a noticeable effect. Optimum HA levels were achieved at 30 °C after 30 h of fermentation. Ucm [28] and Chen [41] suggested slightly higher temperatures, while other studies indicated that 37 °C was ideal [47, 50]. The incubation time also varied among studies. For example, Kim [50] linked the molecular weight of HA produced to the growth phase of bacterial cells. The decline in HA concentration after 30 h might be attributed to several factors. One possibility is the exhaustion of nutrients in the medium, which can hinder the production of HA. Another potential reason is the production of extracellular enzymes that degrade HA over time. It's also plausible that as the microbial growth reaches the stationary phase, the cells start utilizing HA as a carbon source, leading to its degradation. Additionally, changes in environmental conditions like pH and the accumulation of toxic metabolites during fermentation might also affect HA stability and production. Investigations into these mechanisms will provide a clearer understanding. Furthermore, our results indicate that an agitation speed of 180 rpm was ideal for *K. pneumoniae* H15. Previous studies showed a range in optimal speeds, from 170 rpm [16, 51–54] up to 1200 rpm [50]. Faster agitation, however, might negatively affect HA quality due to high shear rates causing potential damage to the HA polymer [48, 55]**.** While it is generally believed that a higher agitation rate can help in releasing HA capsules from bacterial cells into the medium, other factors come into play. At a very high agitation rate (like 250 rpm), the sheer stress can potentially damage the structure of HA, leading to a decrease in its concentration. Additionally, high agitation rates can cause oxygen to be introduced faster into the system, potentially leading to the production of reactive oxygen species (ROS) which can damage the HA. Conversely, at lower agitation rates (like 50 rpm), the environment might be more conducive for the bacterial cells to produce and release HA without causing damage to its structure. It's crucial to strike a balance to optimize both cell growth and HA release. Further studies are required to determine the most appropriate agitation rate and its influence on HA biosynthesis and release. A notable difference in HA concentration was observed between aerated and anaerobic cultures. Higher aeration rates enhanced the production of HA [48, 56]. However, while some studies reported no impact from aeration rate changes [50]**,** others emphasized the balance between lactate synthesis from glucose and the rate of HA synthesis, especially at low agitation speeds [48]. The differential yield in HA concentrations between aerated and anaerobic fermentation cultures can be attributed to several factors: 1. Oxygen as a Growth Promoter: Many bacterial strains involved in HA production thrives better under aerobic conditions. Oxygen can promote better bacterial growth, which in turn can lead to increased HA synthesis. 2. Metabolic Pathways: The presence of oxygen can alter the metabolic pathways used by the bacteria. In aerobic conditions, certain pathways might be favored that lead to the synthesis and accumulation of HA as opposed to other metabolic products that might be produced under anaerobic conditions. 3. Enhanced Nutrient Uptake: Aeration can ensure that nutrients in the medium are uniformly available and can enhance the uptake of essential nutrients, which can be a limiting factor in HA production. 4. Optimal pH Maintenance: Aeration can assist in maintaining a favorable pH during fermentation, particularly because it can offset the acidification of the medium due to the production of organic acids. An optimal pH is critical for enzymatic processes involved in HA synthesis. 5. Cell Membrane Permeability: Aerobic conditions might influence the permeability of the bacterial cell membrane, facilitating the release of HA into the culture medium. Sugar is crucial for the glycolytic pathway. It not only provides energy but also serves as a precursor for HA biosynthesis [17].

Sugar plays a pivotal role in the glycolytic pathway, not only providing energy but also serving as a precursor for HA biosynthesis [17]. Our results demonstrate that among various carbon sources, sucrose (7.0%, w/v) was the most effective, yielding the highest HA concentrations. This finding aligns with Gedikli [17] discovery that sucrose (7.0%) resulted in superior HA production by S. *equi.* Similarly, Pan [47] found sucrose and glucose as the optimal carbon sources for *S. zooepidemicus* ATCC 39920. The use of sucrose in fermentation also appears to enhance the molecular weight of HA [57]. Multiple studies have also highlighted the suitability of sucrose and glucose as primary carbon sources for HA production [13, 41, 58–61]. The study's data further reveals that a combination of yeast extract and peptone serves as the best nitrogen source for HA production. However, when used individually, yeast extract still produced substantial HA concentrations. Similarly, Gedikli [17] reported that yeast extract–casein peptone mixture was the most effective N source in HA production yielding 0.5 g HA L^−1^ by *S. equi*. Pan [47] stated that the highest HA production was obtained upon using yeast extract as N source recording 0.534 g HA L^−1^ in a medium containing glucose and 0.592 g HA L^−1^ in a medium containing sucrose. Khue and Vo [61] showed that yeast extract was found to be the best N source for HA production among different sources. Though the combined mixture of yeast extract–peptone produced the maximum concentrations of HA, however no significant differences (*P* ≥ 0.05) in HA concentrations were obtained upon utilizing yeast extract as N source. Hence, it is advisable to utilize yeast extract as a sole N source in HA production based on the economic point of view. Finally, the cytotoxic impact of HA from *K. pneumoniae* H15 on three cancer cell lines (MCF-7, HepG-2, and HCT) was examined through the MTT assay. The results showed a dose-dependent cytotoxic effect: higher HA doses resulted in reduced cancer cell proliferation. Gedikli [17] had similar findings, observing varying degrees of cytotoxicity on different cells. Gedikli [17] observed varying HA cytotoxicity levels: minimal on THP-1 cells and most pronounced on Human vascular endothelial cells (HUVEC). They also noted that HA concentrations of 3 and 6 mg mL-1 were cytotoxic to human dermal keratinocyte cells (HaCaT) and L929 cells, while a 1.5 mg mL-1 concentration was non-toxic to these cells. Our study determined the IC50 of HA for HCT, MCF-7, and HepG-2 cells to be 273.4, 1538, and 1209 µg mL^−1^, respectively. The variation in IC50 values can be attributed to factors such as HA concentration, incubation duration, and cell type. Additionally, while some studies suggest high molecular weight (MW) HA promotes cell division, others find it cytotoxic [62, 63]. In scientific literature, the effects of high molecular weight (MW) hyaluronic acid (HA) on cells have been diverse. Some studies have shown that high MW HA can promote cell division, suggesting potential benefits in tissue regeneration and repair. On the other hand, there are also findings indicating that high MW HA can be cytotoxic to certain cell types, meaning it can lead to cell death. The precise mechanisms underlying these contrasting effects are still under investigation. In the context of our study, understanding the differential effects of HA on cell behavior is crucial, especially when evaluating its potential therapeutic applications and safety profiles. While our study offers an in-depth examination, some limitations and avenues for future exploration should be highlighted: 1. Optimization Techniques: Our predominant use of the "one variable at a time" approach has its strengths, but it doesn't capture potential interactions between different factors, which might lead to misinterpretations. Adopting statistical optimization methods like fractional factorial design and response surface methodology could be beneficial. These techniques, as showcased in studies by Lotfy [64], Walid [65], and El-Helow [66], can better elucidate the intricate interplays between variables, offering a more rounded perspective on the subject. 2. Delving into Interactions: Using the above statistical approaches will assist in revealing nuanced interactions between variables, crucial for fine-tuning HA production and refining the fermentation process. 3. Broadening the Microbial Scope: Though our study concentrated on *K. pneumoniae* H15, exploring a wider range of microbial strains could offer more insights. Research, like that by Ghanem [67], emphasizes the benefits of studying a diverse set of organisms to enhance our grasp on biotechnological processes. In light of the above, the logical progression in this research field entails incorporating rigorous statistical methods and broadening the microbial exploration. This strategy is poised to deepen our comprehension of the underlying mechanisms and optimize HA production efficiency. Furthermore, Understanding the pathogenicity and virulence of the *K. pneumoniae* H15 strain remains challenging. As we contemplate large-scale HA production, comprehending the potential health risks associated with this strain is essential. Future research priorities should delve deeper into *K. pneumoniae* H15's potential pathogenic traits, especially in the context of upscaling HA production. Another area of concern is the potential contamination of HA with endotoxins, particularly when produced in a system containing *K. pneumoniae* H15. A promising approach to mitigate this concern is to explore the production of HA through a cell-free system. Not only does this offer a potentially cleaner production method, reducing endotoxin contamination risks, but it might also lead to increased yields and reduced purification costs. Our future endeavors will focus on establishing and optimizing such a cell-free system for HA production. Our study, while shedding light on novel methods of HA production, also underscores the importance of a holistic approach that considers both efficiency and safety. We believe that addressing these limitations and integrating the aforementioned future perspectives can drive the next wave of advancements in microbial HA production.

## Conclusion

Overall, this study highlights the characterization of the most favorable cultural conditions for HA production by a promising isolate, *K. pneumoniae* H15 recovered from animal feces. The findings contribute to expanding the knowledge on HA production using microbial fermentation and provide valuable insights for industrial applications of HA in pharmaceutical, medical, biomaterial, and cosmetic fields. Further research and scale-up studies are warranted to explore the commercial potential of *K. pneumoniae* H15 for HA production and its downstream applications in various industries.

## Materials and methods

### Samples collection

A total of 108 samples from different sources were collected from April to December 2020. The samples included 30 samples from cultivated soil with lettuce (at 15 cm depth), 23 samples from animals excretions (10 samples from fresh feces after disinfecting the anal area with 70% ethanol and 13 samples from urine), 25 samples from different foodstuffs collected from shops in Zagazig, Egypt (beef burger, vegetable salad, chicken pane and Shawarma) and 30 samples were collected from patients in Zagazig University Hospital (15 samples from patients' blood and 15 samples from patients' wounds contaminated with pus). All samples were collected in sterile plastic bags and were immediately transferred aseptically in ice box to Bacteriology Lab for isolation.

### Bacterial isolation

The bacterial isolates were recovered from the samples collected by serial dilution technique, where one gram of each sample type (soil, food, and feces) was separately suspended in 9 mL of sterile saline (8.5 g NaCl in 1000 mL distilled water) and vortexed vigorously for 15 min to obtain suspension. Each suspension was serially diluted from 10^–1^ to 10^–9^ [68]. Spread plate method was used to isolate the bacteria form the diluted samples where 0.1 mL from each dilution was pipetted out onto plates with nutrient agar and spread with a sterile cotton swab. A loop of urine and blood samples and wound swabs were directly streaked onto nutrient agar plates [69]. For the clinical blood samples, to prevent coagulation and to maintain their integrity, they were drawn into tubes containing an anticoagulant, such as EDTA. It's worth noting that while blood is typically sterile, there are instances of bacteremia or bloodstream infections in conditions like severe pneumonia, endocarditis, or sepsis. Considering this, blood samples were initially enriched in BHI broth for duration to amplify any bacterial presence. Post-enrichment, these samples were streaked onto blood agar plates for further isolation. All plates were incubated at 37 °C for 24 h and the developed bacterial colonies were purified on BHI agar plates using the streaking method to obtain a pure single colony. These were then preserved on BHI slants after incubation at 37 °C for 24 h. Slant cultures were subsequently maintained at 4 °C.

### Screening of bacterial isolates for HA production

#### Basal medium used in HA production

A total of 108 bacterial isolates were screened for HA producing ability by cultivation on medium recommended by Güngör [13] which composed of (g L^−1^): sucrose (80 g), peptone (10 g), yeast extract (28 g), K_2_SO_4_ (0.5 g), MgSO_4_ (1.25 g), FeSO_4_ (0.008 g), NaCl (1.23 g), and trace elements solution (26 mL composed of CaCl_2_, 2 g; ZnCl_2_, 0.046 g; CuSO_4_.5H_2_O 0.019 g per liter), pH adjusted to 7 by using 1N NaOH and 1N HCl before sterilization. 50 mL of the prepared medium was dispensed into 250 mL Erlenmeyer flasks and autoclaved (steam sterilizer BK-75–01 autoclave-Tomen, Russia) at 121 °C and 1.5 atm for 15–20 min.

#### Inoculum preparation and cultivation conditions

Trypticasein soy broth (TSB, Lanxess Co, Germany) was used as the seed culture by separately inoculating sterilized test tubes containing 5-mL TSB by bacterial isolates followed by incubation at 37 °C for 24 h in a shaking incubator Labtech, Model: LSI-3016A / LSI-3016R, under agitated conditions at 125 rpm. One mL of the inoculum prepared was inoculated in the Erlenmeyer flasks containing the production medium which were then incubated at 37 °C for 24 h at 180 rpm.

#### Extraction and separation of HA from bacterial cultures

HA extraction was performed according to the method described by Brown [70], in which 50 μL of 10% sodium dodecyl sulfate solution (SDS, 100 g in 1 L) was added separately to the bacterial culture followed by incubation at room temperature for 10–15 min with shaking for 2 min then 2 mL of 10% cetyltrimethylammonium bromide (CTAB, 100 g in 1 L) was added to Erlenmeyer flasks which were incubated at 10 °C for 1 h followed by centrifugation at 4000 rpm for 20 min. The pellets were solubilized in 10 mL of 2 M CaCl_2_, left for 6 h at 4 °C, centrifuged at 4000 rpm for 20 min, and the supernatant was taken and 2 volumes of absolute ethanol was added followed by incubation for 1 h at 4 °C. After which, centrifugation was employed at 4000 rpm for 20 min and the pellet was solubilized in 10 mL bi-distilled water for overnight at 4 °C with shaking in order to dissolve the pellet. Centrifugation was performed at 4000 rpm for 20 min and the supernatant was collected and equal volume of 1% NaCl was added followed by extraction with 2 volumes of absolute ethanol. The solution was centrifuged at 4000 rpm for 20 min and the pellet was then solubilized in 5 mL bi-distilled water for overnight at 4 °C. The step of centrifugation and solubilization of the pellet was repeated and the resultant supernatant was filtered with nitrocellulose mixed ester syringe filters (0.45 µm) and the extracted sample was stored at 4 °C for further analysis.

### Quantification of HA

#### Carbazole method

Carbazole-sulfuric acid method was used as a colorimetric determination based on Cesaretti [71]. Two reagents were prepared in this method; reagent1 (4.77 g disodium tetraborate in 500 ml sulfuric acid) and reagent 2 (0.125 g carbazole in 100 mL absolute methanol). Sample (1 mL) in test tube was placed in ice water and cooled to 4 °C. Freshly prepared reagent 1 (5 mL) was added to the test tube, which was then capped, shaken, and placed in a boiling water bath (95–100 °C) for 15 min before being cooled to room temperature. Reagent 2 (0.2 mL) was added to the test tube and put again in a boiling water bath (95–100 °C) for 15 min. After this, they were cooled to room temperature and transferred to Enzyme Linked Immunosorbent Assay (ELISA) plate and the absorbance (color) of the solutions was read at wavelength 530 nm using a Jenway Genova spectrophotometer (UK) against the blank sample in the plate reader [72]. The average optical density of the sample reading was calculated, and the HA concentration was determined from a standard curve.

#### Turbidimetric method

Two reagents were prepared for this HA determination method based on Oueslati [73]: reagent 1 (2 g NaOH in 100 mL distilled water) and reagent 2 (2.5 g CTAB in 100 mL reagent 1). Sample (1 mL) was added in test tube and incubated at 37 °C for 15 min then 2 mL of reagent 2 was added followed by incubation at 37 °C for 10 min and shaking for 10 S at the beginning and at the end of this incubation. The sample was transferred to ELISA plate and the absorbance (turbidity) of the solutions was read at 600 nm against the blank sample using a Biotek Synergy HT Microplate Reader (USA). The average optical density for each reading of samples was calculated and the HA concentration in the sample was determined from HA standard curve [73]**.**

#### HPLC method

A chromatography (Young Lin 9100 instrument, YL 9100, Anyang, South Korea) coupled with UV Spectrophotometric detector and equipped with a vacuum degasser (YL9101), a quaternary pump (YL9110), a column thermostat (YL9131), a diode array detector (YL9160), an auto sampler (YL9150), and software Clarity (version 7.3, Data Apex) was used for all measurements. Chromatographic separations of HA were done by a Great Smart RP18 Aq (150 × 4.6 mm, 3 μm) column (Columbia, USA). The column C18 was tempered at 35 °C. The packing injection volume was 5 μL. The mobile phase consisted of H_2_O: acetonitrile (96:4) (V/V) is freshly prepared and degassed with ultrasound for 30 min and kept in a tightly closed bottle [35]. The freeze-dried HA standard with MW of 1500–1750 KDa (Sigma-Aldrich) was used to prepare HA standard by dissolving 0.5 g of HA in 1000 mL distilled water. The solutions were kept in refrigerator at 4 °C overnight to obtain a homogenous solution. The flow rate of mobile phase was adjusted to 0.7 mL min^−1^. The diode array detector was operated at wavelength 205 nm. Chromatograms were evaluated according to retention times and UV spectra of standard and samples [35].

#### Morphological and cultural characterization of HA-positive isolates

The superior bacterial isolate for HA production was subjected to Gram staining. Its cultural growth was observed on BHI agar and blood agar plates by streaking a loopful of fresh isolate culture onto the two medium and incubation at 37 ˚C for 24 h.

#### VITEK MS identification

Positive bacterial isolates for HA production were identified by using Matrix-assisted laser desorption ionization–time of flight mass spectrometry (MALDI-TOF MS) with a Vitek MS system from biomeriux (France). The identification was based on the VITEK® MS V3.3 Knowledge Base database. A portion of pure colony taken from fresh cultures was spread on the MALDI-TOF MS slide [74]. The bacterial smear was mixed with 1 mL of the matrix solution (an aromatic organic acid consisted of cyano-4-hydroxycinnamic acid in 2.5% trifluoroacetic acid and 50% acetonitrile) and the target slide was left for 5 min at room temperature until dryness and co-crystallization before placing the slide for further analysis. After that, the sample and the matrix were exposed to a laser beam to be ionized. The sample's analytes are removed and ionized by the laser beam. The produced peptide mass fingerprints (PMF) were processed by the engine of the computer and then the spectrum classifier algorithm identifies the bacteria automatically by comparing the PMF of the tested organism with the PMF included in the reference database spectra.

### Molecular identification of the HA most active isolate

#### DNA Extraction

Genomic DNA extraction from the most potent HA-producing isolates was performed at the Animal Health Research Institute, Giza, Egypt, using the QIAamp DNA Mini Kit from QIAGEN. The process of DNA extraction and PCR amplification was carried out according to the manufacturer's instructions.

#### Primers and PCR Conditions

Prior to sequencing, the rRNA gene was amplified using PCR technique in which two universal primers were incorporated in the reaction mixture according to Lagacé [75]. The primers used for gene amplification have the following composition: F27 (AGAGTTTGATCMTGGCTCAG) and R1492 (TACGGYTACCTTGTTACGACTT). A volume of 25 μL reaction mixture was prepared in a 0.5 mL PCR tubes as follows: 12.5 μL Emerald Amp GT PCR master mix (2 × premix), 4.5 μL PCR grade water, 1 μL Forward primer, 1 μL Reverse primer, 6 μL Template DNA. The PCR amplification was carried out with adjusting the cycling conditions of the primers: one cycle primary denaturation at 94˚C for 5 min; 35 cycles with a 30 s secondary denaturation at 94 ˚C for 30 secs, annealing at 56˚C for 60 s and extension at 72˚C for 60 s. The purified PCR products (amplicons) were reconfirmed using a size nucleotide marker (100–1500 bp) by electrophoresis on 1% agarose gel. These bands were then eluted by using QIAquick gel purification kit*.* (Qiagen Inc. Valencia CA) and sequenced by using a ready reaction Bigdye Terminator V3.1 cycle sequencing kit (Perkin-Elmer/Applied Biosystems, Foster City, CA), with Cat. No. 4336817. Sequence homologous was identified in the National Center for Biotechnology Information (NCBI) and GenBank database using Basic Local Alignment Search Tool (BLAST) programs [76]. Multiple alignments and phylogenetic interpretations were made using CLC Bio Sequence Viewer (version 7.7, Qiagen Aarhus, Denmark). The sequence (1485 bp) was deposited in the GenBank with accession number (OP354286).

### Effect of cultural conditions for HA production

#### Effect of physical parameters on HA production

To determine the optimal physical conditions for HA production, a comprehensive study was conducted to evaluate the impact of various physical parameters. Incubation Temperature: The influence of temperature on HA production was studied by incubating bacterial cultures at temperatures ranging from 25 °C to 45 °C. The HA production medium recommended by Gedikli [17] was used for each temperature setting, and subsequent to incubation, the medium was analyzed to determine the HA concentration. Initial pH: The role of pH in HA production was assessed by adjusting the production medium's initial pH using 1N HCl or 1N NaOH, spanning a pH range from 5 to 9. After these adjustments, the cultures were incubated and later evaluated for HA production. Fermentation Period: An exploration was conducted to identify the optimal fermentation duration. Cultures were allowed to grow in the HA production medium across time intervals ranging from 12 to 36 h, in 6-h increments. At the end of each interval, the medium was analyzed to measure HA levels. Agitation Speed: Understanding the potential impact of agitation on HA production, its effect was assessed by incubating cultures at speeds ranging from 50 to 250 rpm. The HA medium, as described by Gedikli [17], was used for this purpose. Following the incubation, the medium was sampled to ascertain the HA concentration.

#### Effect of nutritional parameters on HA production

To assess the most favorable carbon source for HA production, different sugars were incorporated into the basal medium. Glucose, lactose, maltose, and fructose were individually substituted for sucrose in the basal medium at concentrations of 70 g L^−1^. The cultures were then incubated under predetermined optimal physical conditions. Post-incubation, the HA concentrations in the cell-free supernatants were analyzed. A parallel culture with the basal medium containing sucrose was maintained as the control for a comparative assessment [77]. The impact of varying nitrogen sources on HA production was also explored. Different nitrogenous compounds, namely peptone, yeast extract, a combination of yeast extract and peptone, ammonium sulfate, and sodium nitrate, were each incorporated into the basal medium at concentrations of 25 g L^−1^. Following the incubation under the recommended physical parameters, the cell-free supernatants were analyzed for HA concentrations. A culture with the basal medium that contained a standard or commonly used nitrogen source was set up as the control for this evaluation [77].

### Cytotoxicity effects of HA

#### Preparation of different concentrations of HA

A stock solution of HA separated from *K. pneumoniae* H15 cultures (10 mg HA were separated from the bacterial cultures, as described earlier in the section of extraction and separation of HA) was prepared. Different concentrations of HA solution were adjusted, as follows: 500, 250, 125, 62.5, 31.25, 15.63, 7.81, 3.91, 1.95, 0.98, 0.49, and 0.24 µg mL^−1^ via dilution with distilled water in order to investigate their cytotoxic activities against cell lines in vitro.

#### Cell culture and maintenance

Cell lines, viz., breast cancer cell line (MCF-7), human liver cancer cell line (HepG-2), and colon cancer cell line (HCT), were obtained from the American Type Culture Collection (ATCC, Rockville, MD, USA) and cultured in Dulbecco’s modified Eagle’s medium (DMEM) supplemented with necessary growth factors. The subculture was performed after reaching 90% confluence. The growth media were renewed every day.

#### Measurement of cytotoxicity test

The IC50 (half maximal inhibitory concentration) represents the concentration of HA required to reduce cell viability by half relative to the untreated controls. For its calculation, we used an MTT assay, which evaluates cell metabolic activity. Post-exposure to HA, the degree of reduction in cell viability directly relates to the compound's cytotoxic effects. Initially, cells were seeded on a 96-well microplate at a density of 1 × 10^4^ cells in each well and then incubated at 37 °C. After 24 h, once the confluence reached approximately 90%, the old culture media were replaced with fresh ones. Following this, the cells were treated with varying concentrations of HA, keeping certain rows as untreated controls. Following incubation intervals of 24, 48, and 72 h at 37 °C in an environment with 5% CO2, potential morphological alterations in the cells were scrutinized microscopically. Thereafter, MTT solution was introduced to each well. Subsequent incubation allowed for the conversion of MTT to formazan crystals within viable cells. These crystals were later solubilized using DMSO or 0.4% acidic isopropanol. Absorbance of each well was then measured at 570 nm using an ELISA plate reader (Biotek Synergy HT Microplate Reader (USA**) **[78]**.** The IC50 value, representing the concentration at which there is a 50% inhibition, was calculated using dose–response curve analysis. The data obtained from the cytotoxicity tests were plotted with concentrations on the X-axis and the respective percentage inhibition or cell viability on the Y-axis. The curve was then generated using nonlinear regression analysis. The IC50 value was determined from the point on the curve where the response was reduced by half. Cell viability was calculated employing the formula sourced from Wang [79]: Cell viability percentage (%) = (OD of treated cells / OD of untreated cells (control) × 100.

To determine the inhibition percentage, we employed the formula as suggested by Lotfy [80]: Inhibition rate (%) = ((OD control group − OD test group)/ OD control group) × 100.

### Statistical analysis

Data were processed using MS Excel (Microsoft Corporation, Redmond, WA, USA).

We assessed normality and homogeneity of variance using the Levene and Shapiro–Wilk tests [81]. The impact of different treatments was analyzed using a one-way ANOVA (PROC ANOVA), with a significance threshold set at α = 0.05. Results are presented as mean values ± SE. Graphs were generated using GraphPad Prism software 5.0 (GraphPad, USA). A p-value below 0.05 was considered statistically significant.

## Data Availability

The datasets utilized and/or examined in the present study can be obtained by contacting the corresponding author. Additionally, the genetic sequence of the strain analyzed has been submitted to the GenBank nucleotide sequence database at the National Library of Medicine, National Center for Biotechnology Information (NCBI). The assigned accession number for the sequence is OP354286, which can be accessed at https://www.ncbi.nlm.nih.gov/nuccore/OP354286.
